# Women and Men’s Perspectives on the Factors Related to Women’s Dyadic Sexual Desire, and on the Treatment of Hypoactive Sexual Desire Disorder

**DOI:** 10.3390/jcm10225321

**Published:** 2021-11-15

**Authors:** Enav Friedmann, Julie Cwikel

**Affiliations:** 1 Department of Business Administration, Guilford Glazer Faculty of Business and Management, Ben Gurion University of the Negev, POB 653, Beer Sheva 84105, Israel; EnavFrie@bgu.ac.il; 2 Center for Women’s Health Studies and Promotion and Spitzer, Department of Social Work, Ben Gurion University of the Negev, POB 653, Beer Sheva 84105, Israel

**Keywords:** sexual desire of women, hypoactive sexual desire disorder (HSDD), gender differences, help-seeking, biopsychosocial model of sexuality

## Abstract

Sexuality is a basic human need, which is expressed in the context of intimate personal relations. However, in studies of women’s sexuality, men’s attitudes are often overlooked. Health care providers can benefit from the examination of how both women and men perceive women’s sexual desire and what are the most acceptable avenues for treatment for women’s hypoactive sexual desire disorder (HSDD). This research aimed to explore differences between women and men on the factors affecting women’s sexual desire and the appropriate avenues for treatment. Data were collected using an online questionnaire from 233 heterosexual adults who had a dyadic, steady intimate relationship over most of the previous year. A theory-based questionnaire of 28 items was developed to explore the factors associated with women’s sexual desire. One quarter (7/28) of the items affecting women’s sexual desire were ranked significantly differently between women and men. Among women, interpersonal issues and physical attraction, and among men, physical attraction and daily hassles were the significant predictors of women’s sexual desire. Women more than men endorsed psychological help such as a sex therapist or psychologist as a more appropriate treatment for HSDD, while both men and women viewed the internet as a reasonable way to gain treatment information. Both women and men viewed gynecologists as a more acceptable source of treatment than a family doctor. Religious authorities were the least likely source of treatment advice for both women and men. The results support a multi-dimensional model of women’s sexual desire and suggest that psychological interventions to treat HSDD may be preferred by women more than men.

## 1. Introduction

Sexual desire is defined as a motivational state to engage in sexual activities or an interest in a sexual partner, or partners and common sexual activities [1]. Sexual desire can be dyadic (interest in engaging in sexual activity with another person) or solitary (interest in engaging in sexual behavior by oneself) [1]. In this research, we focus on dyadic sexual desire that consists mainly of wanting to engage in sexual activity in relation to a partner [2]. We further explore the factors that are identified by women and men as affecting, both positively and negatively, women’s sexual desire and how men and women feel that women should receive treatment for reduced sexual desire.

Female sexual dysfunction (FSD) is characterized by decreased sexual desire, decreased arousal, limited orgasmic function and often pain during sexual intercourse [3,4,5,6]. A narrower term, hypoactive sexual desire disorder (HSDD), relates specifically to the elements of desire, interest in sex or libido [7]. Brotto defined HSDD as a persistent or recurrent deficit of sexual fantasies and desire for sexual activity in concordance with the DSM-IV-TR (Diagnostic and Statistical Manual of Mental Disorders—4th edition, Text Revision) [4]. This was the definition used in the current study.

Psychosocial factors that negatively affect HSDD include communication difficulties, sexual misinformation, destructive or abusive past or present relationships and poor sex education [8,9]. Negative cognitive and emotional factors include anxiety about sexual performance, distracting thoughts about bodily appearance and flashbacks from earlier traumatic sexual encounters that interfere with sexual arousal and pleasure [10,11]. A qualitative study suggested that both men and women similarly conceptualize sexual desire as a subjective, psychological experience, but women emphasize a more interpersonal and emotional basis for sexual activity compared with men [12].

A theoretical, biopsychosocial model proposed by Basson posits that women’s sexual response is more than a physical experience and is determined by psychosocial factors, specifically the need for a relationship [13]. This need leads women to be receptive to sexual advances, or to initiate sexual play with their partner, generating sensual stimuli that, in turn, drive sexual arousal, which then reinforces a sense of togetherness and relationship commitment. Toates proposed a theoretical integrative framework that emphasized both positive and negative valences that either enhance or inhibit sexual desire, arousal and behavior, but without empirical validation [14].

Different measures of FSD (which includes HSDD), based on different definitions, have shown varying prevalence rates among women, and have also differed in the population under study [15,16]. For example, a comprehensive review showed that premenopausal women reported significant rates (20–30%) in community studies [4]. A similar rate was reported in a sample of 14,480 women in Israel aged 18 and older (28%) [17]. Studies have tended to report inflated rates in “normal” populations (19–50%), with rates increasing among those with sexual complaints (68–75%) and being as high as 79% among post-menopausal women in Turkey [18]. A study published in *JAMA* by Laumann and colleagues found that decreased female sexual desire over the life course was reported, in answer to a single question, by 43% of women [19]. Shifren et al. found that current low desire was reported by 39% of women, in a large community sample in the USA, again in response to a single question [20].

As HSDD incorporates different biological, psychological and contextual factors that influence women’s sexual desire, these factors may be assessed differently by men and women. Several studies have found that men are more focused on frequent and intense episodes of sexual drive than women [21,22,23]. Some studies have claimed that gender differences in sexual activity are context-dependent [23,24]. For example, Petersen and Hyde found these differences are smaller in cultures that have more gender equity [25]. Nonetheless, the majority of research in this area portrays a clear gender difference regarding processing sexual content: men process erotic information and words faster than women do, while women have a more complex organization and memory of relationship-oriented words [26]. Women’s sex drive is primarily energized by emotions connected with intimate relationships so that women tend to describe their sexuality as satisfying when they have a close relationship with their partner [27,28,29].

As women are considered more aware of cues from the environment and are more socially connected, they are also found to seek advice or assistance from others more frequently than men [30,31]. Specifically, men have been found to be more reluctant to seek help from health professionals compared to women when facing diverse problems such as depression, substance abuse, physical disabilities and stressful life events [32,33]. Both men and women are increasingly turning to internet sites for advice on sexual issues and dysfunction, but it is not clear whether there is a significant gender difference in the use of these sources of assistance [34,35,36].

The present study attempts to bridge the knowledge gaps presented above by exploring the full range of factors that influence women’s sexual desire, in both a positive and negative fashion. We included men in this study for several reasons: they are often the medical policy makers and women’s physicians and therapists, and they influence the acceptability of medical treatments in general and the help-seeking patterns of their female partners. The literature up until now has rarely included men’s perceptions of what shapes women’s sexual function, desire and how HSDD should be treated [1,7,37,38]. Some scholars have suggested that not all women regard decreased sexual desire as a problem requiring treatment [22,39].

Specifically, we hypothesized:

H1: Women and men will differ on the factors they view as important in determining women’s sexual desire so that the interpersonal dimension will be a more significant factor for women than men.

H2: Women compared to men will think it is more appropriate to ask for help from psychological treatment sources compared to medical treatment sources when dealing with HSDD.

## 2. Materials and Methods

### 2.1. Sample

Survey data were collected from 233 people, 77 men and 156 women, in Israel. Respondents answered an online Qualtrics survey through a link that was distributed via Facebook, email and Whatsapp. The project was presented as a “study of women’s sexual desire” and was limited to heterosexual adults over the age of 18 who had a steady intimate partner for most of the past year. Respondents were directed to the online survey, where they read the explanation and agreed to an informed consent statement. If the respondents did not meet the inclusion criteria, then the survey terminated. The research design was approved by the department Ethics Committee.

The average age of men in the sample was 43.17 years (SD =11.023, minimum 27, maximum 75), and the average age of women was 39.11 years (SD = 10.128, minimum 21, maximum 69) (age difference: t(226) = −2.745, *p* = 0.007) (see Table 1). Most of the respondents (69%) were married, with 14% living with a partner, 11% single and 6% divorced or widowed. A majority of persons in the sample had children (71%); the mean number of children was 2.59 (SD = 1.17). The mean years of education was 18.7 years (SD = 4.19). The sample was mostly secular (78%), while 15% defined themselves as traditional and 7% religious. Mean self-rated health was 6.13 out of a 7-point scale (SD = 1.11). With regard to income, 2% did not agree that their family’s income covers their basic needs, 6% somewhat agreed, 13% did not agree or disagree, 27% somewhat agreed and 52% completely agreed.

### 2.2. Procedure

The questionnaire was developed in two stages. In the first stage, a literature search was conducted and a list of variables that affect women’s sexual desire was generated from current research [4,16,19,40,41,42,43]. We did not find a full scale that included the variables that shape sexual desire in the literature and that took a broad biopsychosocial approach including both positive and negative effects. Afterwards, the list was evaluated for face validity by five judges who are professionals working in the area of sexual treatment and research, who were asked to indicate if these variables indeed affect sexual desire and to suggest additional variables if needed. This generated a list of 28 variables (see Table A1).

Based on these results, a descriptive survey was constructed. The questionnaire included questions about: perceptions of different variables that can affect sexual desire, how often women want to engage in sex, where women with HSDD should go for treatment, as well as standard demographic questions (gender, age, education, religion, religiosity and income) (see Supplementary Materials).

### 2.3. Measures

All measures about sexuality and sexual function were asked indirectly about women in general, to overcome embarrassment and discomfort. Third person, indirect questions that assumed that the respondent is answering from his/her personal experience are especially appropriate in researching “sensitive” issues [44]. Direct questioning is vulnerable to social desirability bias, particularly for questions that ask about intimate, personal topics. For Likert-scaled answers, anchoring wording was given only on the extremes of the scales (1 or 7), with 4 designated as the median or neutral state.

**Variables affecting the sexual desire of women**—participants were asked to rate the influence of the 28 variables on the sexual desire of women. A score of 1, 2 and 3 reduces sexual desire very much (1), quite a bit (2) and somewhat (3), 4 is neutral, and 5, 6 and 7 represent a variable that increases sexual desire (somewhat, quite a bit, very much, respectively). These included: psychosexual and biological variables and interpersonal and contextual issues (see Table A1). Cronbach’s alpha was 0.66.

**Sexual desire was measured by one item based on Shifren** [20]—how often do you think women want to engage in sexual activity?

The answering options were: (1) not at all, to (7) a lot (several times a day).

**Help seeking—** participants were asked to suppose a woman has no interest in sex for several months and rate how appropriate it was to seek help from each of the eight sources based on Jones [45]. These sources included: family physician, gynecologist, sex therapist, family or friends, reading on the internet, religious authority, psychologist and family/couples therapist. All were measured on a 7-point Likert scale ranging from (1) not at all appropriate to (7) very appropriate.

### 2.4. Statistical Analyses

We examined the mean of all variables from the questionnaire that might influence the sexual desire of women according to men and women, as shown in Table A1. Given that there were significant differences between women and men on some of these variables, we continued to analyze women and men separately. Furthermore, we analyzed the sample in two age cohorts: women and men up to age 40 and over age 40. These age-specific results are shown in Table A2 and Table A3. Factor scores were created for women and men (from the full sample) that were later used as independent variables in linear regression analyses when predicting sexual desire. The factor analysis was predetermined to extract three dimensions on the assumption that factors would group into positive, neutral and negative influences on sexual desire. For example, open communication with the partner should have a positive influence on sexual desire, drinking alcohol/cold weather was expected to be neutral as it may increase or decrease sexual desire, and everyday hassles should have a negative influence. Linear regression was also used to examine whether the factor scores, derived for women and men separately, predicted women’s sexual desire, separately for each gender.

The eight sources for treatment were factor analyzed, and then independent t-tests were conducted to check for possible gender differences in the suitability of different sources of support to deal with HSDD. Last, a repeated-measures, mixed (between-within) ANOVA analysis was conducted, where gender was a between-subject factor predicting the appropriateness of the three treatment sources (within). This analysis was to test for a possible interaction between sources of treatment for HSDD and gender.

## 3. Results

It is interesting to see the different perceptual world of men and women when exploring variables that relate to women’s sexual desire. Table 2 presents the results.

According to men in the sample, the negative dimension of “daily hassles” appeared first in explaining the sexual desire of women. By contrast, the positive physical–interpersonal aspect was the first factor that affects women’s sexual desire according to women respondents. This aspect included communication and meaningful talk as a combined experience of attraction and closeness, whereas among men this dimension was split into two factors (communication and attractiveness). It is interesting to note which factors were included in women’s factors but not in men’s and vise-versa. For women, imagination and foreplay were part of the first, positive factor, but were not included in men’s views. Little time together loaded on the third, negative factor for women but was not included in men’s results. Fights, attention, alcohol, winter and housework variables loaded only in the men’s results.

The factor analysis enabled us to see what variables have common variances but did not help us to determine what variables were perceived as more influential in women’s sexual desire, according to gender. For this purpose, regression analyses were performed for men and women separately, predicting sexual desire to test H1. The specific factors of men and women were entered as predictors to predict women’s sexual desire. The results are presented in Table 3.

As seen in Table 3, among women, when predicting women’s sexual desire, only the interpersonal and physical attractiveness factor remained significant in predicting sexual desire, while for men, daily hassles and physical attractiveness had a significant impact, while the interpersonal dimension was not significant. However, in the assessment by women, the factors were more interdependent: the only significant factor that predicts women’s sexual desire was the interpersonal–physical dimension, suggesting physical attractiveness was related to open communication with the partner and having meaningful talks.

For H2, we first analyzed the descriptive results of each support source, which are presented in Table 4. Differences between men and women were found only concerning psychological sources of advice (therapists, psychologists and consulting with family and friends), with women more likely to use these sources than men. No differences were observed in seeking help from other sources (family physician, gynecologist, internet, religious authority) (see Table 4).

We then examined the different treatment sources in a factor analysis (see Table 5).

As can be seen from Table 5, the first factor was more psychological and informal, including sex therapist, family/couple therapist, psychologist, using the internet and consulting with family and friends. The second medical factor consisted of a gynecologist and family physician, and the last factor was a single variable of seeking advice from a religious authority.

**Figure 1:**
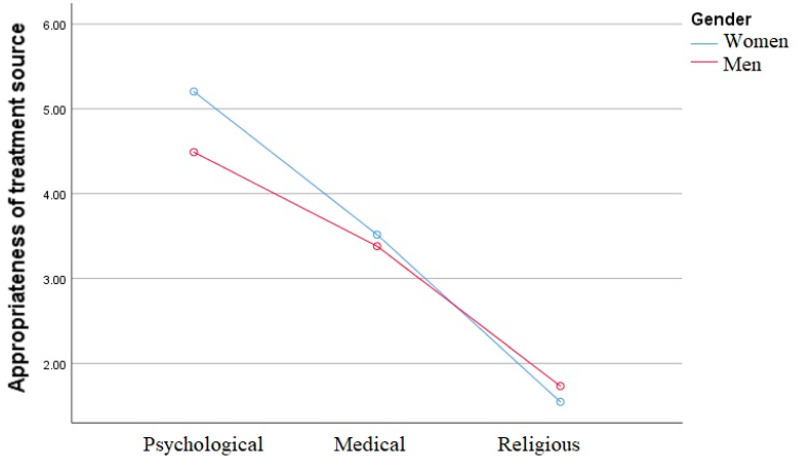
Interaction between gender and appropriateness of treatment sources. Note: Main effect of treatment source (F (2,220) = 392.49, *p* < 0.001), interaction between gender and treatment source (F (2,220) = 8.07, *p* < 0.01).

When examining the significance of the gender differences regarding the factors of the sources, we found that the main effect of treatment source was significant (F (2,220) = 392.49, *p* < 0.001), and also the interaction between gender and treatment source was significant (F (2,220) = 8.07, *p* < 0.01), as demonstrated in Figure 1.

Even though both genders view psychological treatment as more appropriate to deal with HSDD than medical treatment, gender differences appeared only when evaluating the appropriateness of the psychological treatment source (*d* = 0.85, t_224_ = 4.47, *p* < 0.001), and not when evaluating the appropriateness of the medical treatment source (*d* = 0.12, t_224_ = 0.51, *p* = 0.31).

## 4. Discussion

The objective of this study was to explore perceptions of the factors the affect women’s sexual desire as perceived by women and men. Overall, the results of this study supported Toate’s theoretical view that both positive and negative aspects affect sexual desire. H1 assumed that among women the interpersonal dimension would be more significant when predicting women’s sexual desire, and this was confirmed [14]. However, the variables that comprised the interpersonal factors were different for women and men and the relationship between the factors and women’s sexual desire was also different.

Women in our study saw physical attractiveness and communication as the foremost dimensions influencing their sexual desire. It is especially interesting that these two dimensions of physical attractiveness and communication were connected for women and were separate independent dimensions in men’s perceptions. Men ranked everyday hassles as the most important dimension detracting from sexual desire for women. This may be explained in three ways: (1) everyday hassles have reduced their own libido, hence they assume it affects women’s sexual desire; (2) men in this study may have wanted to have more frequent sexual liaisons with their partners and felt that daily hassles frequently detracted from their partner’s sexual desire; or (3) they have expressed an empathic response, indicating that they recognize the environmental and contextual factors that influence female desire.

Physical attractiveness (of both partners) was the second ranked predictor of women’s sexual desire in the men’s regression analysis. It is interesting that when men evaluated what influences women’s sexual desire, they did not attribute any major significance to communication or other interpersonal factors.

Age influenced the importance of some factors, as older women and men (above 40) gave greater importance to attention from the partner, compared with younger women and men. Older women gave greater importance to cold weather and over-awareness of body image, while younger women gave higher importance to alcohol and days close to the monthly menstruation cycle. Older men were more likely to report a negative influence of fatigue, worries over the security situation and medical problems of one of the partners compared to younger men.

H2 examined the gender differences in the appropriateness of seeking help from different sources when dealing with HSDD. H2 was partially confirmed as women thought it was more appropriate to ask for help from psychological authorities such as a sex or family therapist or a psychologist or to seek support from family/friends than men did. In light of the H1 results that found the interpersonal and physical attraction is a connected factor for women, this finding suggests that women regarded the treatment of a sexual problem as more a psychological–interpersonal problem.

However, when looking at the appropriateness of each source to treat HSDD, no differences were found between men and women regarding medical help. In one study, women were more likely to seek psychological help for matters that distressed them than men, and this is in accordance to what we found in our research [46]. While the women in this study were close to age 40 on average, it is interesting that in a study of women over age 50, only one-quarter actually discussed sexual issues with their physician [47]. This reticence to discuss sexual issues with physicians is common. However, women have also been socialized that it is awkward to discuss sexual concerns with their partners. In a recent review of qualitative studies on the sexual behavior of older women, it was found that both spiritual beliefs and long-held notions about what is proper sexual behavior were significant in shaping how these women expressed sexuality in their intimate relationships [48]. This may explain why, in our results, using the internet to obtain information on sexual matters and particularly sexual dysfunction was favorably regarded by both women and men.

Overall, the results emphasize the importance of the interpersonal context in interaction with their partner as shaping how women view their sexual desire. The importance of communication with a partner as a key factor affecting women’s sexual desire thus supports the biopsychosocial model as proposed by Basson [13]. This suggests that a medical treatment model for HSDD would not meet the needs of many women.

One relatively simple medical treatment is to provide a drug to treat HSDD among women. Indeed, recreating the phenomenal success, both medically and economically, of Viagra (*Sildenafil*) has been a long-term drug industry goal [7,38,49]. However, treatments developed for women such as the testosterone patch, Viagra prescriptions for women and testosterone gel have repeatedly failed to gain Food and Drug Administration (FDA) approval [7,49,50]. Recently, the FDA gave approval to a drug (Vyleesi) to treat HSDD, which requires injection 45 minutes before sexual intercourse. However, its effectiveness is barely greater than the placebo treatment and the drug has significant detrimental side effects such as nausea and darkening of the skin and gums [51]. Another drug, *Flibanserin,* approved by the FDA for HSDD not associated with any co-morbid medical problem, also has significant side effects, needs to be taken on a daily basis and is dangerous to mix with any alcohol consumption [52]. A meta-analysis of 10 trials concluded that *Flibanserin* did not differ significantly from placebo treatments [53]. Thus, drug treatments for women’s HSDD that have a reasonable cost–benefit balance are still elusive. It is reasonable that women do not want to risk significant side effects and the hassles of systemic drug treatments or injections in order to treat a problem that may be a matter of communication and amenable to psychological treatment. This may explain why psychological treatments were seen as a better avenue for treatment than medical sources for women in our study.

This research has some limitations. It examined a heterosexual Western population using an internet survey that does not necessarily represent the whole population, particularly as some persons do not have ready access to the internet. As this was an exploratory study, we limited the sample to heterosexuals in order to create a more uniform sample. However, future research should include respondents of a range of sexual orientations. Thus, it does not represent the views of LBGT individuals or those who live in polyamorous relationships. These populations should be included in future studies.

It was not a clinical sample, which would have included only persons who are currently troubled by low sexual desire, but our sample is congruent with our objective to explore perceptions of what influences women’s sexual desire and the appropriate source for treatment. It is imperative to replicate this study with other populations in order to ascertain whether the results are consistent across countries and cultures. This could be accomplished by using recently validated questionnaires that look both at the psychological aspects of women’s sexuality and view sexuality in the context of relationships and interpersonal stress among both men and women, e.g., [54,55]. Furthermore, in our study, the questions were asked indirectly to avoid social desirability bias and respondent inconvenience when asking them directly about sexual attitudes and behaviors. Future research could examine this directly, as stereotypical beliefs about female sexual function might influence their answers.

## 5. Conclusions

Nonetheless, this research adds to the evidence on the differences between the genders on the perceptions of sexual desire among women currently living in an intimate relationship, and the ways it is appropriate to seek help for problems associated with decreased sexual libido. This is particularly important in the medical management of older women and men, when the physician or therapist needs to be especially sensitive to the context of decreased sexual desire and function [48,55]. This suggests that a biopsychosocial, contextual, integrated model of women’s sexual desire should guide treatment, both for women and their partners and for health care professionals.

## Figures and Tables

**Table 1:** Demographic characteristics of the sample.

	Women *n* = 156		Men *n* = 77		Total *N* = 233	
	Mean	SD	Mean	SD	Mean	SD
Age *	39.11	10.128	43.17	11.023	40.41	10.571
Education *	8.711	3.588	19.74	5.126	18.7	4.19
Income (“my family status covers my basic needs”)						
Did not agree	2%		1.4%		2%	
Somewhat disagreed	7.8%		1.4%		6%	
Did not agree or disagree	13.7%		12.2%		13%	
Somewhat agreed	24.8%		32.4%		27%	
Completely agreed	51.6%		52.7%		52%	
Family Status						
Married or living with partner	78.9%		84.4%		80.7%	
Single	13.5%		6.5%		11.2%	
Widowed, divorced, separated	7.6%		9.1%		8.1%	
With children *	67.3%		79.2%		71%	
Mean number of children	2.53	1.074	2.69	1.329	2.59	1.17
Mean self-rated health	6.14	1.183	6.09	0.939	6.13	1.11
Religiosity						
Secular	79.9%		74.3%		78%	
Traditional	14.3%		16.2%		15%	
Religious	5.8%		9.5%		7%	

Significant differences between men’s and women’s demographics: * age difference: t(226) = −2.745, *p* value = 0.007; * education level difference: t(224) = −2.596, *p* value = 0.01; * with children difference: χ^2^(1) = −5.127, *p* value = 0.016.

**Table 2:** Factor analysis on what influences the sexual desire of women, by gender.

**Women (Total Variance Explained 37.9%)**			
	**Factor 1—“Interpersonal and Physical** **Attractiveness”**	**Factor 2—“Daily Hassles”**	**Factor 3—“Security and Little Time Together”**
Little time together *			0.575
Attractive partner	0.753		
Woman’s feeling of attractiveness	0.740		
Stress		0.814	
Fatigue		0.838	
Hassles		0.800	
Child care		0.713	
Mood	0.689		
Memory of past relationships	0.578		
Imagination *	0.654		
Security			0.545
Foreplay *	0.726		
Meaningful talks	0.713		
Open communication	0.720		
Variance explained	18.5%	12.2%	7.2%
**Men (Total Variance Explained 34.4%)**			
	**Factor 1—“Daily Hassles”**	**Factor 2—“Interpersonal”**	**Factor 3—“PhysicalAttractiveness”**
Fights **	0.690		
Attention **		0.635	
Alcohol **		−0.510	
Winter **			0.545
Attractive partner			0.606
Woman’s feeling of attractiveness			0.565 *
Stress	0.821		
Fatigue	0.786		
Hassles	0.805		
Child care	0.655		
Memory of past relationships			0.654
Security		−0.564	
Meaningful talks		0.722	
House work **		0.591	
Open communication		0.595	
Variance explained	16.7%	10.03%	7.7%

* Variable loadings above 0.5, only in the women’s factor analysis; ** Variable loadings above 0.5, only in the men’s factor analysis.

**Table 3:** Regression estimates predicting women’s sexual desire by factors by gender, separately.

		B Coefficient	t Value	Exact *p* Value
Women	Intercept	4.869	54.785	0.000
	F1—”interpersonal and physical attractiveness” **	0.343	3.851	0.000
	F2—“hassles” **	0.153	1.711	0.089
	F3—“security and time together”	0.136	1.526	0.129
R^2^	0.131			0.000
Men	Intercept	4.540	33.832	0.000
	F1—“hassles” **	0.370	2.751	0.008
	F2—“interpersonal”	0.051	0.385	0.702
	F3—“physical attractiveness” *	0.321	2.286	0.026
R^2^	0.188			0.007

* *p* < 0.05; ** *p* < 0.01.

**Table 4:** Appropriate treatment source for HSSD—gender differences (scale 1–7, 7 very appropriate).

Profession	Women		Men		
	Mean	SD	Mean	SD	Exact *p* Value
**Sex therapist**	5.92	1.57	5.32	1.60	0.009
**Family or couple therapist**	5.56	1.51	4.79	1.71	0.001
Internet	5.38	1.56	5.13	1.69	NS
**Psychologist**	5.18	1.72	4.04	1.87	0.001
Gynecologist	4.54	2.24	4.12	1.95	NS
**Family or friends**	4.03	2.04	3.15	1.96	0.003
Family doctor	2.47	1.71	2.77	1.70	NS
Religious figure	1.55	1.25	1.73	1.43	NS

Note: NS = not significant. Variables listed in **bold** differed significantly between women and men.

**Table 5:** Factor analysis of appropriate sources for treatment.

Overall Variance Explained 65.6%			
	Factor 1—“Psychological”	Factor 2—“Medical”	Factor 3—”Religious”
Sex therapist	0.627		
Family or couple therapist	0.852		
Internet	0.621		
Psychologist	0.781		
Gynecologist		0.890	
Family or friends	0.607		
Family doctor		0.733	
Religious figure			0.797
Variance explained	34.97%	17.75%	12.89%

Note: Five items were averaged to represent the psychological factor, and two to represent the medical factor, in later analysis.

## Data Availability

The data can be obtained by contacting the first author, E.F.

## Supplementary Materials

The following are available online at https://www.mdpi.com/article/10.3390/jcm10225321/s1, Supplementary File: The Study Questionnaire.

## Appendix A

**Table A1:** Mean values of the variables that affect women’s sexual desire rated by men and women.

Men		Women
1	**When the woman feels attractive (6.406)**	Feeling of love from the partner (6.62)
2	Feeling of love from the partner (6.250)	When the woman feels attractive + (6.577)
3	Foreplay (6.188)	**When the partner is attractive + (6.395)**
4	**When the partner is attractive (6.156)**	Foreplay (6.234) and open communication (6.234)
5	Open communication (6.094)	Good mood (6.139)
6	Good mood (6.031)	**Getting attention from the partner + (6.117)**
7	**Getting attention from the partner (5.74)**	**Meaningful discussions with the partner + (6.036)**
8	Creative and active imagination (5.688)	Creative and active imagination (5.920)
9	**Meaningful discussions with the partner (5.406)**	Memories from past intimate relationships (5.577)
10	Memories from past intimate relationships (5.234)	Drinking alcohol (4.978)
11	Drinking alcohol (4.953)	Doing household chores together (4.832)
12	Watching pornography together (4.828)	Watching pornography together (4.766)
13	Cold weather (4.734) and doing household chores together (4.734)	Cold weather (4.715)
14	Over-awareness of body image (4.297)	**Days close to the monthly menstruation cycle + (4.299)**
15	Oral contraceptives (4.172)	Over-awareness of body image (3.942)
16	**Days close to the monthly menstruation cycle (3.875)**	Oral contraceptives (3.832)
17	The possibility that a pregnancy may result (3.859)	The possibility that a pregnancy may result (3.496)
18	Lack of time with partner (3.172)	Lack of time with partner (3.117)
19	Worries over the security situation (3.063)	Worries over the security situation (2.825)
20	Medical problems with one of the partners (2.609)	Medical problems with one of the partners (2.613)
21	**Knowing that the woman will not reach orgasm with her partner (2.578)**	**Knowing that the woman will not reach orgasm with her partner − (2.285)**
22	Arguments with her partner (2.391)	Care of children (2.102)
23	**Partner who is focused on meeting his own needs (2.375)**	Hard to ignore thoughts that sex is forbidden or dirty (2.080)
24	Hard to ignore thoughts that sex is forbidden or dirty (2.297)	Arguments with her partner (2.029)
25	Care of children (2.125)	Everyday stress (1.891)
26	Everyday stress (1.948)	**Partner who is focused on meeting his own needs − (1.818)**
27	Daily hassles (1.844)	Daily hassles (1.825)
28	Fatigue (1.594)	Fatigue (1.642)

Note: +**Bold** font indicates a significant difference between men and women; +: more positive; −: more negative.

We recoded the value to be 1 to 7, so a positive effect on desire is from 5–7, neutral is 4 and 1–3 decreases desire, so that, for example, sexual attractiveness, foreplay and love increase sexual desire, and stress, hassles and fatigue decrease sexual desire.

Only 7 variables were ranked differently between men and women. Four out of those 7 variables are related to interpersonal relationship.

Women gave higher positive values to:

1. Attention from the partner (Mean = 6.15 SD = 1.274) than men (Mean = 5.77 SD = 1.129), t = 2.106, *p* = 0.036.

2. Attractive partner (women: Mean = 6.40, SD = 1.013; Men: Mean = 6.07, SD = 0.865, t = 2.447, *p* = 0.015).

3. Attractive women (women: Mean = 6.59, SD = 0.963; Men: Mean = 6.29, SD = 0.874, t = 2.309, *p* = 0.022).

4. Meaningful talk with the partner (women: Mean = 6.00, SD = 1.153; Men: Mean = 5.42, SD = 1.303, t = −2.964, *p* = 0.003).

5. Days near the menstrual cycle had a relatively positive influence on women while it had a relatively moderate influence in the eyes of men (women: Mean = 4.30, SD = 1.553; Men: Mean = 3.88, SD = 1.343, t = 2.010, *p* = 0.046).

Two variables were perceived as having a stronger negative influence on the sexual desire of women, by women when compared to men:

1. Partner being selfish (women: Mean = 1.84, SD = 1.323; Men: Mean = 2.40, SD = 1.299, t = −2.964, *p* = 0.003).

2. Knowing that the women will not reach orgasm (women: Mean = 2.25, SD = 1.202; Men: Mean = 2.69, SD = 1.204, t = −2.562, *p* = 0.011).

## Appendix B

**Table A2:** Mean values of the variables that affect women’s sexual desire by younger women and men (below 40) *.

Men (*n* = 28)		Women (*n* = 85)
1	When the woman feels attractive (6.37)	When the woman feels attractive (6.69)
2	Feeling of love from the partner (6.18)	**When the partner is attractive + (6.50)**
3	Foreplay (6.07) and **When the partner is attractive (6.07)**	Feeling of love from the partner (6.47)
4	Good mood (5.89)	Foreplay (6.33)
5	Open communication (5.86)	Open communication (6.23)
6	Creative and active imagination (5.61)	Good mood (6.15)
7	**Getting attention from the partner (5.32)** and Memories from past intimate relationships (5.32)	**Meaningful discussions with the partner + (6.06)**
8	Drinking alcohol (5.18)	**Getting attention from the partner + (5.92)**
9	**Meaningful discussions with the partner (5.14)**	Creative and active imagination (5.88)
10	Watching pornography together (4.75)	Memories from past intimate relationships (5.44)
11	Cold weather (4.71)	Drinking alcohol (5.26)
12	Doing household chores together (4.57)	Doing household chores together (4.73)
13	**The possibility that a pregnancy may result (4.54)**	Watching pornography together (4.70)
14	Days close to the monthly menstruation cycle (4.22)	Days close to the monthly menstruation cycle (4.55)
15	Over-awareness of body image (4.07)	Cold weather (4.50)
16	Oral contraceptives (3.96)	Oral contraceptives (3.74)
17	Worries over the security situation (3.50)	Over-awareness of body image (3.52)
18	Medical problems with one of the partners (3.07)	**The possibility that a pregnancy may result − (3.49)**
19	Lack of time with partner (2.93)	Lack of time with partner (3.08)
20	Arguments with her partner (2.71)	Worries over the security situation (2.85)
21	Knowing that the woman will not reach orgasm with her partner (2.68)	Medical problems with one of the partners (2.64)
22	**Partner who is focused on meeting his own needs (2.57)**	Arguments with her partner (2.33)
23	Care of children (2.26)	Knowing that the woman will not reach orgasm with her partner (2.21)
24	Hard to ignore thoughts that sex is forbidden or dirty (2.14)	Hard to ignore thoughts that sex is forbidden or dirty (2.02)
25	Everyday stress (2.11)	Care of children (2.00)
26	Daily hassles (1.96)	Everyday stress (1.96)
27	Fatigue (1.75)	**Partner who is focused on meeting his own needs − (1.82)** and Daily hassles (1.82)
28		Fatigue (1.64)

* One woman and four men did not indicate their age and thus their data are missing. Note: +**Bold** font indicates a significant difference between men and women; +: more positive; −: more negative. We recoded the value to be 1 to 7, so a positive effect on desire is from 5–7, neutral is 4 and 1–3 decreases desire.

Women gave different values to:

1. Attention from the partner (women: Mean = 5.96, SD = 1.37; men: Mean = 5.32, SD = 1.42), t = 1.97, *p* = 0.05).

2. The possibility that a pregnancy may result (women: Mean = 3.49, SD = 1.52; Men: Mean = 4.54, SD = 1.77, t = −3.02, *p* = 0.003).

3. When the partner is attractive (women: Mean = 6.50, SD = 0.93; Men: Mean = 6.07, SD = 0.77, t = 2.21, *p* = 0.03).

4. Partner who is focused on meeting his own needs (women: Mean = 2.57, SD = 1.55; Men: Mean = 1.82, SD = 1.34, t = −2.48, *p* = 0.02).

5. Meaningful discussions with the partner (women: Mean = 6.06, SD = 1.15; Men: Mean = 5.14, SD =1.49, t =3.40, *p* = 0.01).

## Appendix C

**Table A3:** Mean values of the variables that affect women’s sexual desire by older women and men (40 and over) *.

Men (*n* = 45)		Women (*n* = 69)
1	Feeling of love from the partner (6.36)	Feeling of love from the partner (6.62)
2	When the woman feels attractive (6.27)	When the woman feels attractive (6.47)
3	Open communication (6.18)	**Getting attention from the partner + (6.39)**
4	Foreplay (6.11)	When the partner is attractive (6.28)
5	Good mood (6.04)	Foreplay (6.21)
6	**Getting attention from the partner (6.02)**	Good mood (6.07)
7	Creative and active imagination (5.62)	Meaningful discussions with the partner (5.93)
8	Meaningful discussions with the partner (5.58)	Creative and active imagination (5.88)
9	**Memories from past intimate relationships (5.20)**	**Memories from past intimate relationships + (5.75)**
10	Watching pornography together (4.86)	Cold weather (4.97)
11	Doing household chores together (4.76)	Watching pornography together (4.82)
12	Cold weather (4.71)	Doing household chores together (4.78)
13	Drinking alcohol (4.55)	Drinking alcohol (4.74)
14	Over-awareness of body image (4.27)	Over-awareness of body image (4.41)
15	Oral contraceptives (4.22)	Oral contraceptives (4.06)
16	Days close to the monthly menstruation cycle (3.69)	Days close to the monthly menstruation cycle (4.00)
17	The possibility that a pregnancy may result (3.32)	The possibility that a pregnancy may result (3.52)
18	Lack of time with partner (3.24)	Lack of time with partner (3.25)
19	Worries over the security situation (1.82)	Worries over the security situation (2.93)
20	Knowing that the woman will not reach orgasm with her partner (2.64)	Medical problems with one of the partners (2.62)
21	Medical problems with one of the partners (2.40)	Knowing that the woman will not reach orgasm with her partner − (2.28)
22	Hard to ignore thoughts that sex is forbidden or dirty (2.34)	Care of children (2.19)
23	Arguments with her partner (2.31)	Hard to ignore thoughts that sex is forbidden or dirty (2.16)
24	Partner who is focused on meeting his own needs (2.27)	Arguments with her partner (2.03)
25	Care of children (2.09)	Partner who is focused on meeting his own needs (1.88)
26	Stress (1.82)	Daily hassles (1.83)
27	Daily hassles (1.67)	Stress (1.80)
28	Fatigue (1.42)	Fatigue (1.55)

We recoded the value to be 1 to 7, so a positive effect on desire is from 5–7, neutral is 4 and 1–3 decreases desire. * One woman and four men did not indicate their age and thus their data are missing. Note: +**Bold** font indicates a significant difference between men and women; +: more positive; −: more negative.

Women gave a higher positive influence to:

1. Attention from the partner (Mean = 6.32, SD = 1.10) than men (Mean = 6.02, SD = 0.81), t = 1.93, *p* = 0.05.

2. Memories of past intimate relationship (women: Mean = 5.75, SD = 1.22; Men: Mean = 5.20, SD = 1.32, t = 2.26, *p* = 0.03).

To conclude what the differences are between the age groups, we further explored the difference between the ages among women and men separately and found:

Older women (above 40) gave a higher influence compared with younger women to:

1. Attention from the partner (older: Mean = 6.39, SD = 1.10; younger: Mean = 5.92, SD = 1.37), t = 2.32, *p* = 0.02.

2. Cold weather (older: Mean = 4.97, SD = 1.51; younger: Mean = 4.50, SD = 1.43), t = 1.98, *p* = 0.05.

3. Over-awareness of body image (older: Mean = 4.41, SD =1.78; younger: Mean = 3.52, SD = 1.87), t = 2.94, *p* = 0.004.

While younger women (below 40) gave a higher influence compared with older women to:

1. Alcohol (older: Mean = 4.74, SD = 1.69; younger: Mean = 5.26, SD = 1.54), t = −1.20, *p* = 0.05.

2. Days close to the monthly menstruation cycle (older: Mean = 4.00, SD = 1.39; younger: Mean = 4.55, SD = 1.65), t = −2.17, *p* = 0.03.

Older men (above 40) gave a higher influence compared with younger men to:

1. Attention from the partner (older: Mean = 6.02, SD = 0.82; younger: Mean = 5.32, SD = 1.42), t = 2.69, *p* = 0.009.

2. Fatigue (older: Mean = 1.42, SD = 0.54; younger: Mean = 1.75, SD = 0.80), t = 1.98, *p* = 0.05.

3. Worries over the security situation (older: Mean = 2.82 SD = 1.00; younger: Mean = 3.50, SD = 1.11), t = 2.09, *p* = 0.04.

4. Medical problems with one of the partners (older: Mean = 2.40, SD = 1.03; younger: Mean = 3.07, SD = 0.98, t = 2.76, *p* = 0.007.

While younger men (below 40) gave a higher influence compared with older men to:

1. The possibility that a pregnancy may result (older: Mean = 3.32, SD = 1.34; younger: Mean = 4.54, SD = 1.77), t = 3.31, *p* = 0.001.
