# Supplementary material for: Women and Men’s Perspectives on the Factors Related to Women’s Dyadic Sexual Desire, and on the Treatment of Hypoactive Sexual Desire Disorder
Source: J Clin Med. 2021 Nov 15;10(22):5321. doi: 10.3390/jcm10225321 (PMC8623679; doi:10.3390/jcm10225321)
Supplement: Supplementary file 1 [file jcm-10-05321-s001.zip › jcm-1405525-supplementary.pdf]

The Study Questionnaire

This survey investigates the dynamics of women's sexual desire, and is designed for both heterosexual men and women over the age of 18 and who are currently in a relationship that has lasted for at least three months. The survey is anonymous; you cannot be identified from your answers. The results will be used solely for academic research. The survey takes about 10 minutes. By filling in the survey, you have shown your agreement to participate in this project. Thank you for your honest and insightful answers.

Screening questions:

What is your age? (please write in years) _____ (screened for >18)

What is your sexual orientation? Gay/ Bisexual/Transexual/Heterosexual (screened for Heterosexual)

Did you have a steady, intimate partner for most of the past year? Yes/ No (screened for Yes)

Please rate the influence of the following factors on women's sexual desire. Mark -3, -2, -1 if the factor reduces women's sexual desire and 1, 2, 3 if it increases women's sexual desire. A score of -3 reduces sexual desire very much (-3), quite a bit (-2), somewhat (-1), 0 is neutral, and 1, 2, 3 represent an increase in sexual desire (somewhat, quite a bit, very much), respectively.

	-3 Reduces sexual desire	-2	-1	0 Neither increases nor decreases	1	2	3 Increases sexual desire
Lack of time with her partner	○	○	○	○	○	○	○
Thinking that sex is forbidden or dirty	○	○	○	○	○	○	○
Feeling love for her partner	○	○	○	○	○	○	○
Arguments with her partner	○	○	○	○	○	○	○
Getting attention from her partner	○	○	○	○	○	○	○
Drinking alcohol	○	○	○	○	○	○	○
The possibility that a pregnancy may result	○	○	○	○	○	○	○
Cold weather	○	○	○	○	○	○	○
When the woman thinks her partner is attractive	○	○	○	○	○	○	○
When the woman feels attractive	○	○	○	○	○	○	○
Every day stress	○	○	○	○	○	○	○
Fatigue	○	○	○	○	○	○	○
Daily hassles	○	○	○	○	○	○	○
Childcare	○	○	○	○	○	○	○

Good mood	○	○	○	○	○	○	○
Memories from past intimate relationships	○	○	○	○	○	○	○
Worries over her body image	○	○	○	○	○	○	○
When her partner is mainly focused on meeting his own needs	○	○	○	○	○	○	○
Creative and active imagination	○	○	○	○	○	○	○
Worries over the security situation	○	○	○	○	○	○	○
Foreplay	○	○	○	○	○	○	○
Meaningful discussions with her partner	○	○	○	○	○	○	○
Oral contraceptives	○	○	○	○	○	○	○
The days just before her menstrual cycle	○	○	○	○	○	○	○
When the woman knows she will not reach orgasm with her partner	○	○	○	○	○	○	○
Medical problems of one of the partners	○	○	○	○	○	○	○
Watching pornography together	○	○	○	○	○	○	○

Doing household chores together	○	○	○	○	○	○	○
Open communication with her partner	○	○	○	○	○	○	○

How often do you think women want to engage in sexual activity?

(1) Not at all (2) Once a month (3) Once in two weeks (4) Once a week (5) Twice a week (6) Several times a week (7) A lot (several times a day).

Suppose a woman has no interest in sex for several months. Rate how appropriate it would be to seek help from each of the eight sources (where (1) Not at all appropriate to (7) Very appropriate).

	1 Not at all appropriate	2	3	4	5	6	7 Very appropriate
Sex therapist	○	○	○	○	○	○	○
Family or couple therapist	○	○	○	○	○	○	○
Internet	○	○	○	○	○	○	○
Psychologist	○	○	○	○	○	○	○
Gynecologist	○	○	○	○	○	○	○
Family or friends	○	○	○	○	○	○	○
Family doctor	○	○	○	○	○	○	○
Religious figure	○	○	○	○	○	○	○

Are you:

○ Married or living with a partner

○ Single

○ Other ____

Do you have children?

○ Yes

○ No

How many children do you have?

____

What is your level of religiosity ?

- ○ Religious
- ○ Traditional
- ○ Secular

How do you rate your health?

- ○ 1 Very poor
- ○ 2
- ○ 3
- ○ 4
- ○ 5
- ○ 6
- ○ 7 Very good

How many years of education have you completed?

____

Please state your agreement to the following: My family’s income covers my basic needs

- 1 Do not agree
- 2 Somewhat disagree
- 3 Do not agree or disagree
- 4 Somewhat agree
- 5 Completely agree
